# CADE-Q SV: Practical and Relevant in the Assessment of Patients with Cardiovascular Diseases regarding their Health Condition

**DOI:** 10.5935/abc.20180236

**Published:** 2018-12

**Authors:** Juliana Beust de Lima

**Affiliations:** Universidade Federal do Rio Grande do Sul - Programa de Pós-Graduação em Ciências da Saúde: Cardiologia e Ciências Cardiovasculares, Porto Alegre, RS - Brazil

**Keywords:** Cardiovascular Diseases/mortality, Health Education, Survey and Questionnaires, Physical Fitness, Cardiac Rehabilitation, Exercise, Prevention and Control

Cardiovascular diseases (CVD) remain the leading cause of death worldwide. According to estimates by the World Health Organization (WHO), approximately 17.9 million individuals die per year due to this clinical condition.^1^ In Brazil, a registry of the Brazilian Society of Cardiology shows an increase in mortality over the years, affecting 383,961 individuals in 2017.^2^

Considering the strong association of CVD with morbidity and mortality, as well as the impairment of functional capacity and quality of life shown by these patients, strategies aimed at minimizing these impairments and that can be shown to be cost-effective should be implemented. In this scenario, cardiovascular rehabilitation (CVR) is crucial and should be part of the overall treatment of CVD.^3^

The WHO defines CVR as "the sum of activities required to ensure people with CVD have the best possible physical, mental and social conditions, so that the patients may, by their own efforts, preserve or resume when lost as normal a place as possible in the society."^4^ According to the South-American Guideline for Cardiovascular Prevention and Rehabilitation,^3^ for this process to be feasible and for the objectives to be attained, the integrated performance of a multidisciplinary team is required. Physical training, associated with drug treatment, is the central component of CVR. However, the professionals' performance is not restricted to creating the plan and applying the exercises. Special attention should also be given to the patients' full education regarding their health condition and appropriate management of risk factors, aiming at having a healthy lifestyle.^3^ Specific characteristics of each patient, such as socioeconomic and educational levels, may influence the prior knowledge and understanding of information provided by professionals. In this sense, tools that evaluate the patients' knowledge regarding their health condition are useful and can help the professionals to create and apply effective strategies.

Recently, the validation of the Portuguese version of the Coronary Artery Disease Education Questionnaire - Short Version (CADE-Q SV) was published in this journal.^5^ This questionnaire assesses the knowledge of CVD patients regarding their health condition, including the clinical aspects, risk factors, exercise, diet and psychosocial risk. Concerning the previously validated questionnaires (CADE-Q and CADE-Q II),^6,7^ the new proposal maintains the assessment of central components of CVR, with differences being related to the fact that it is a more concise tool that can be applied in ten minutes, as reported by the researchers. The short time required, and the objective profile of the response options may favor the applicability of this tool in research and clinical practice, providing relevant information for better targeting of interventions in secondary prevention in Brazil.

It has been previously shown that the context in which the subject is placed can influence their knowledge about health. In a previous study, using the pioneer CADE-Q version, the researchers found that CVR participants in Brazil had lower levels of knowledge about their condition when compared to Canadian patients.^8^

In the most recent CADE-Q SV validation study, outpatients who did not necessarily participate in a formal CVR program were included. The area identified with greater knowledge was that related to risk factors and the one with the lowest score was that related to psychosocial risk. Overall, the patients showed a low level of knowledge, and those with lower educational level, as well as those with family income, showed significantly higher needs. On the other hand, characteristics such as age younger than 65 years and previous infarction or arrhythmia were associated with significantly higher level of knowledge.^6^

CADE-Q SV is a short, valid and reliable tool to evaluate the knowledge of CVD patients in Brazil. It can be useful in the characterization of groups of patients and especially in the identification of each individual's specific needs, allowing the implementation of targeted educational strategies. Therefore, it is a potential tool to be used in secondary prevention and should be tested and administered in different health programs and regions of our country. After applying the questionnaire, it is recommended that the professionals clarify doubts about the answers to the patients, in order to help in the process of learning and health care.
